# What Role Can Trained Volunteers Add to Chronic Disease Care of Immigrants?

**DOI:** 10.1007/s10903-020-01079-2

**Published:** 2020-09-15

**Authors:** Ellen Rosenberg, Tamara Carver, Nina Mamishi, Gillian Bartlett

**Affiliations:** 1 Department of Family Medicine, McGill University, Montreal, QC Canada; 2 Westmount, Canada

**Keywords:** Health goals, Immigrants, Life goals, Volunteers

## Abstract

To help primary care teams improve patient-centered care, we elicited health and life goals of immigrants with a chronic disease. We conducted an exploratory study of the (1) acceptability of home visits by volunteers to collect health information and (2) content of health and life goals within a primary care program for immigrants with chronic disease. Pairs of trained community volunteers visited 23 patients in their homes and asked them to identify three life goals and three health goals. We conducted content analyses of written notes. Health goals were related to disease prevention and symptom control, family well-being, own quality of life, own or family members’ work and/or financial situation. Life goals concerned family well-being, their own quality of life, work/financial situation and health. Given the limited time health professionals have with their patients, trained community volunteers could be important members of primary care teams caring for immigrants.

## Background

Chronic diseases are one of the most important health care issues affecting Canadians today [1]. To improve the health of people with chronic diseases, professionals have developed disease care guidelines that include treatment goals. Setting goals with patients can improve goal attainment in areas including physical activity, stress management [2, 3], well-being [4], and meeting of targets for chronic conditions, e.g. blood glucose, blood pressure and asthma control [5]. Goals of care for chronic diseases are traditionally set in terms of disease-specific outcomes such as biomarkers and symptoms chosen by health professionals. Both life and health goals, however, are important to patients [6]. For example, the reported goals of patients with low back pain were idiosyncratic to the individual and not necessarily aligned with standard clinical outcome measures [7]. The literature, however, provides evidence of the importance of the integration of life goals into treatment plans. Two studies found that, to be successful in heart failure care, one needed to address patient priority goals first, whether or not they were related to the disease [8, 9].

The potential impact of both health and life goal setting is particularly relevant for immigrants. Many migrant groups of non-Caucasian origins have higher prevalence of Type 2 diabetes mellitus (DM) and cardiovascular disease (CVD) than the host populations in the United Kingdom, Europe, Canada, United States and Singapore [10–12], and lower than average rates of disease control [10, 13]. In addition, migrants have important life goals in their new country such as education and career opportunities for themselves and their children, learning the language, securing housing and employment [14, 15]. Very little work, however, has been done to ascertain health and life goals of immigrants at high risk of chronic diseases.

Goal-setting is a key component of the **Health Teams Advancing Patient Experience: Strengthening Quality (**Health TAPESTRY program) [16]. Health TAPESTRY centers on meeting a person’s health goals with the support of trained community volunteers, technology, an interprofessional team, system navigation, and community engagement. The goals elderly urban Canadians TAPESTRY participants identified most frequently concerned improved productivity, financial planning, travel, and increased physical activity [17]. In our project, AccessHealth, we adapted the Health TAPESTRY program to the needs of immigrants and offered it to immigrants from South Asia (India, Pakistan, Sri Lanka), the Middle East, the Caribbean and the Asia Pacific region who have a chronic illness.

The objective of the project was to assess the feasibility of home visits by volunteers to elicit health information and patient goals. We are reporting our analyses of (1) the acceptability to immigrants of home visits by volunteers, (2) the content of immigrants’ goals, and (3) the numbers and content of goals only elicited by asking for a patient’s life goals.

## Methods

We undertook a project in which trained volunteers elicited patient health and life goals, quality of life, social support, nutrition, and physical activity during a home visit. Each physician received a report of their patient(s)’ responses. The patients were immigrants from South Asia, the Middle East, the Caribbean and the Asia–Pacific region who attended family physicians at one of two academic family medicine clinics in Montreal, Canada. This paper concerns patient reactions to the volunteers and their health and life goal answers only.

### Participants

Adults patients born in South Asia, the Middle East, the Caribbean and the Asia–Pacific region who had at least one of the following chronic diseases: diabetes, obesity, mood disorders/anxiety, chronic disease of the digestive system, hypertension, cancer, and arthritis were invited to participate. Given that the study involved home visits using volunteers, patients with severe cognitive impairment or history of violence, where volunteers might be at risk, were excluded.

### Participant Recruitment

We recruited patients from the practices of family physicians working in two urban family medicine academic group practices in Montreal, Canada. We asked 29 physicians to approach all of their patients from the above-identified regions who had at least one chronic disease; 28 physicians agreed; 13 had eligible patients in their practice. Each physician contributed between 1 and 6 patients. The physicians asked their patients’ permission to have a research assistant contact them to explain the project.

The consent form was obtained by the research coordinator at each clinic and scanned to the volunteer coordinator. Once a scanned copy of the signed consent form was received, the volunteer coordinator contacted the patients to set an appointment for the home visit. Patients were given a copy of their signed consent form at the beginning of the home visit by a pair of trained home visiting community volunteers.

### Volunteer Recruitment and Training

We recruited volunteers through emails to community and health professional groups and posters in community centers and ethnic stores. Volunteers had to have several hours of volunteer experience with ill people and/or minority populations. All the volunteers completed an interview and passed police background and reference checks.

Volunteers completed a set of online training modules developed by the TAPESTRY team through a specially designed virtual learning centre that includes videos, self-assessment quizzes, a learning portfolio, online discussions, and synchronous communication with others. For AccessHealth, we added a cultural competency module using a blended delivery modality. After completing the online modules, volunteers participated in one day of in-person cultural competency training including role-playing and other interactive educational activities.

We trained volunteers to elicit health and life goals using open questions. In this exploratory project volunteers were not integrated into the treatment team. Therefore, we did not train volunteers to guide patients to formulate SMART **(S**pecific, **M**easurable, **A**chievable, **Relevant** and **T**ime-based) goals.

Of the 28 volunteers approached, 19 participated. One volunteer was excluded for unwillingness to follow protocol. Of the 8 who refused, 7 provided a reason: 2 were admitted to medical school, 1 moved to another city, 3 got a full-time job. There were 13 women and 6 men. Their average age was 37.5 years (range 19–66). They were born in Egypt [8], Iran [4], India [3] and one each in Afghanistan, Algeria, Nigeria and Sri Lanka. Eighteen were health professionals: 12 physicians unable to practice medicine in Canada, 1 veterinarian, and 5 nurses.

### Data Collection

The volunteer pair collected information electronically using a tablet computer housing a specifically designed Health TAPESTRY software application (TapApp) which includes structured surveys (quality of life, social support, nutrition, physical activity and acculturation) and semi-structured narratives. In this paper we report only about health and life goals the volunteers elicited through patient narratives. Each section was completed by the patient, facilitated by the volunteer pair. Translation support was provided through over-the-phone translation if needed.

### Measures

#### Health and Life Goals

Volunteers asked patients to provide three life goals. Volunteers described life goals to patients as ‘anything that you may want to maintain, improve, or avoid related to your life, including work, family, or hobbies’. Next, volunteers asked patients to state 3 health goals. Each patient was then asked to identify, from their 6 goals, 3 priority goals: goals to work on in the following 6 months.

### Analysis

We performed content analysis of health and life goals. Our objective was to describe patient goals as expressed in their words. For goals to be likely to be achieved they should be SMART [18]. Therefore, we explored to what extent priority goals produced without guidance were SMART.

We assessed each priority goal using the SMART categories. It was straightforward to identify a goal as specific, e.g. *return to my pre-glaucoma hobbies, meet my endocrinologist to discuss medications*. In contrast, *help my family* is not specific. We classified a not-specific goal as measurable if the goal could be made specific in terms that were measurable, e.g. *be active, exercise, eat healthy*. The difference between attainable and relevant was not immediately clear to us from patient interview content. We have chosen to define an attainable goal as one the person could achieve such as *take my medication* and *go to exercise classes*. In contrast, examples of goals we deemed unattainable are *win the lotter*y and *have a comfortable life without anxiety or stress*. A relevant goal, on the other hand, is a goal that fits with all the other priorities in one’s life. Using this definition, we were unable to assess whether goals were relevant. Therefore, we assessed specificity, measurability, attainability for each goal. All were time-based since the volunteer asked them to name priority goals which were, by definition, to be accomplished within the next six months.

This study was reviewed and approved by the Research Ethics Committee of St. Mary’s Hospital of the Centre integré universitaire de santé et de services sociaux de l'Ouest­de-l'Île-de-Montréal (CIUSSS ODIM).

## Results

Of the 63 possibly eligible patients approached, 54 proved eligible, 31 refused and 23 patients (42.6%) participated. There were 14 women and 9 men. They varied in age from 37 to 91 years, mean 60.5 years, median 55 years. The countries of origin were South Asia (Bangladesh, India, Sri Lanka) 12, Egypt 7, Jamaica 2, Malaysia 1 and Philippines 1. Although each patient was asked to state six goals, patients provided an average of 3 life goals and 3.8 health goals.

### Health Goals

There were 28 goals concerning various aspects of health including disease prevention and symptom control. Four patients simply said to ‘be healthy’ or to ‘stay healthy’ without further specifics. More specific health goals were weight control (4 patients), exercise (6 patients), diet (5 patients), diabetes and/or blood pressure control (8 patients). Two patients aimed to improve their emotional state: ‘Get out from my sickness emotionally’, ‘Manage stress’. Several other goals indicate that patients understand health in a much broader terms than the biomedical. For example, 10 health goals were related to family well-being. There were 4 goals related to the patient’s own quality of life: to ‘entertain myself, ‘enjoy myself’, ‘not to suffer’ and ‘die in peace’. Improving their own or their family members’ work and/or financial situation were the goals of 2 patients. All the health goals of one patient concerned education of children and a better job for the spouse (Table 1).

**Table 1:** Domains of all life and health goals reported by patients

Goals	Health goals	Life goals
	N goals	N goals
Family well-being	10	13
Education of children		
Happiness of children		
Health of relatives		
Work and finances	2	8
Quality of life	4	9
Health	36	20
Being healthy	5	8
Diet	5	1
Exercise	6	5
Weight control	4	3
Symptom control	4	1
Self control of disease	8	1
Specific help from MD	3	0
Emotional	1	1

### Life Goals

Disease prevention and symptom control were the subject of 20 life goals. Patients reported 13 life goals related to family well-being. These goals were most often about their children’s future including education and marriage. There were 9 goals related to patients’ own quality of life. Goals included increasing companionship, hobbies and travel including long visits to their homeland. Eight goals concerned improving their own or their family members’ work and/or financial situation. See details in Table 1.

Eighteen of the 23 patients reported at least one life goal not mentioned as a health goal. For these patients, asking about life goals provided additional information useful to clinicians. These goals appear in Table 2.

**Table 2:** Life goals not elicited as health goals of the same patient

Goal	Number
Work	4
A job, a better job for self or spouse	
Quality of life	4
Companionship	
Hobbies	
Family	12
Health	
Education	
Marriage	
Travel to visit family	
Wife to join family in Canada	

### Priority Goals

When asked to choose from their stated health and life goals 3 goals to work on in the following 6 months, patients identified family wellbeing, personal wellbeing activities, financial and job improvements and health. Of the 23 patients, 13 mentioned at least one priority goal only when asked for life goals (see Table 2).

Of all 68 priority goals, 34% were SMART by our definition, while 12 of the 23 patients (52%) had at least one SMART priority goal. As shown in Table 3, of these 12 patients 6 aimed to control their diabetes, 5 aimed to take their medication and 3 intended to return to previous activities: painting, shopping, piano, singing, hobbies. Stated life goals that were not SMART provided important contextual information of potential use to clinicians as they develop management plans with patients. For example, Mr. M’s priority goals were ‘control diabetes by diet’ (his health goal), ‘find a job’ and ‘live a comfortable life without anxiety or stress’ (his life goals).

**Table 3:** Priority goals = goals patients intended to address in the following 6 months

Priority goals	All goals N	SMART goals N
Family Wellbeing	12	0
Education of children		
Happiness of children		
Health of relatives		
Personal well-being activities	10	6
Companionship		
Hobbies		
Trips		
Maintain or improve health	8	0
Exercise	8	2
Diabetes control	6	6
Diet	6	1
Weight loss or maintenance	5	1
Job for self or spouse/finances	5	0
Take medication	4	4
Specific help from MD	2	2
Effective medication		
Emotional	2	0
Manage stress		
‘Get out from my sickness emotionally’		

## Discussion

There are four main findings of this study. First, immigrant patients are willing to share their goals with trained home visit community volunteers and provide more detail than might be documented in a time-constrained clinical visit. Second, soliciting life goals can yield information important for developing successful patient-centered care plans. Third, goals related to families were very common. Finally, 23% of the goals patients produced spontaneously were SMART.

### Acceptability of Volunteers

In our study 42.6% of immigrant primary care patients who were approached participated which provides information supporting the acceptability to immigrants of volunteers in their home to collect personal information. There are few reports in the literature that look at the acceptability of volunteer home visits [16]. Participation rates in home visit intervention programs by elders varied from 37% in rural Taiwan [19] to 60.4% in Vienna [20]. For some programs to be successful participants must see the volunteers as people who share their values and understand their life contexts. There have been many programs involving Lay Health Advisors to address health disparities in African American populations [21]. Programs have been more successful when the volunteers were similar to the target population in social, cultural, ethnic, and communication values, norms, and beliefs [21]. In many large cities, immigrants do not constitute one sociocultural group. Therefore, matching volunteers to patients of the same group is seldom feasible. Most of our volunteers were first- or second-generation immigrants but they were not from the same sociocultural group as the patients they visited. In this study, the immigrant patients who accepted a home visit, openly shared their life and health goals with volunteers many of whom were quite different from themselves.

### Patient-Reported Life Goals Provide Information Important to Clinicians

Among our 23 patients, 12 had important goals only raised when asked for life goals. The literature provides evidence of the importance of the integration of life goals into treatment and health promotion plans. College students who perceived pursuance of their personal goals as facilitators of physical activity were more physically active as documented by accelerometer [22]. Two studies revealed the importance of addressing life goals of heart failure patients. Zhang et al. asked patients with heart failure to list their top five goals [8]. Symptom control was the primary goal of 25% of patients. The primary goal of 22% of this sick population, however, was a life goal such as taking care of others or spending quality time with family and loved ones [8]. The same authors then indicated the degree to which each heart failure self-care activity, e.g. exercise, was compatible with achievement of any of the patient’s top five goals. Exercise adherence positively correlated with the extent to which the regimen was considered compatible with life goals such as social functioning and autonomy [8]. In another study, home care nurses worked with heart failure patients to set goals and prioritize them. The nurses learned that, to be successful in heart failure care, they needed to address patient priority goals first, whether or not they were related to the disease [9]. Many of the goals concerned family situations, other diseases, and activities that improved their quality of life. Our findings concerning the importance of life goals strengthen the literature on the pertinence of inclusion of patient life goals in chronic disease care.

### Goals Related to Family Have a Prominent Place

Life goals concerning well-being of their families were more numerous and voiced by more of our patients than goals for personal well-being. In contrast, in a study using very similar TAPESTRY methods, no goals relating to family members were mentioned [17]. The personal goals of Australian women with breast cancer include a happy marriage and being there for their families but do not mention children [23].

With that said, the observed prominence of life goals concerning family health and well-being in our study are consistent with new Canadians’ reasons for immigrating to Canada: to improve the future for their family (30%) and to join family or close friends already living in Canada (27%) [15]. Migration adaptation can occupy migrants for many years and the integration into Canada can be a slow process. After 4 years in Canada, immigrant goals still included learning or becoming more fluent in one or both of Canada’s official languages; having their previous education and skills accredited/ recognized and accessing education and training opportunities [24]. Life goals of immigrant patients are affected by their reasons for immigrating, and the social and economic conditions they face as immigrants. The well-being of their children is often of primary importance to them. Therefore, life goals concerning children are often more important to them than any health goals for themselves. This is important information for health care providers to understand the context and limitations of patients to address their own health care concerns.

### Without Guidance, Patients Seldom Express Goals in Terms that Foster Attainment

Most of the stated goals do not lend themselves to application because they were not SMART (**S**pecific, **M**easurable, **A**chievable, **Relevant** and **T**ime-based). Our work has uncovered important weaknesses in our method of eliciting patient life and health goals. Volunteers asked patients to formulate their life and health goals to serve as the focus of patient-centred action plans for health maintenance and chronic disease self-management. Our volunteers, however, posed open-ended questions and provided no guidance. Most resulting patient goals were not specific, measurable or time-based. Only 52% of patients identified at least one SMART goal to work on over the following 6 months. In a study by Parsons and Parsons, before the introduction of a program to help patients receiving an episode of home-care to formulate SMART goals using Towards Achieving Realistic Goal in Elders Tool (TARGET), 8.6% of patients had a goal recorded [24]. Once TARGET was introduced, 94.2% of recipients of an episode of home care had a goal recorded [24]. SMART goals are more likely to be attained [18] when health professionals use TARGET, patients were much more likely to attain their goals than were patients who formulated their goals without guidance (47.8% compared to 2.2%) [24].

As an exploratory study, this work has limitations preventing broad generalizability of the findings. The number of participating patients is not large. The patients are all immigrants in a single Canadian city. The volunteers did not use a procedure to help patients make their goals SMART thus preventing any estimation of the effect of setting SMART goals on patient outcomes.

## Conclusion

Immigrants accepted to share their health and life goals with trained volunteers who came to their homes. For many immigrants, ongoing focus on the health and wellbeing of family members has priority over their own health. Most patients did not express goals in terms that lead to treatment plans and/or measurement of success. Community home visit volunteers can gather information that could contribute to improvement of patient-centered chronic disease care of immigrants.
